# Hope against hope: exploring the hopes and challenges of rural female caregivers of persons with advanced cancer

**DOI:** 10.1186/1472-684X-12-44

**Published:** 2013-12-17

**Authors:** Allison Williams, Wendy Duggleby, Jeanette Eby, Reverend Dan Cooper, Lars K Hallstrom, Lorraine Holtslander, Roanne Thomas

**Affiliations:** 1 School of Geography and Earth Sciences, McMaster University, 1280 Main Street West, Hamilton, ON L8S 4 K1, Canada; 2 Nursing Research Chair Aging and Quality of Life, Level 3, Edmonton Clinic Health Academy, 11405 87 Avenue, University of Alberta, Edmonton, AB T6G 1C9, Canada; 3 Palliative Care Services, Regina Qu'Appelle Health Region, 410w Dewdney Ave, Regina, SK S4T 1A5, Canada; 4 Alberta Centre for Sustainable Rural Communities (ACSRC), Political Studies (Augustana Faculty) and REES (ALES), University of Alberta, 2-135 Augustana Forum, Edmonton, AB T4V 2R3, Canada; 5 College of Nursing, University of Saskatchewan, Office Rm 343 Ellis Hall, 107 Wiggins Rd, Saskatoon SK S7N5E5, Canada; 6 Qualitative Health Research with Marginalized Populations, School of Rehabilitation Sciences, 451 Smyth Road (3068), Ottawa, ON K1H 8 M5, Canada

**Keywords:** Hope, Palliative and end of life care, Caregiving, Rural, Narrative research

## Abstract

**Background:**

This paper focuses on the qualitative component of a study evaluating a hope intervention, entitled Living with Hope Program (LWHP), designed to foster hope in female caregivers of family members living with advanced cancer. The purpose of this research is to share, in the form of a story, the experiences of rural female caregivers caring for family members with advanced cancer, focusing on what fosters their hope. Hope is a psychosocial and spiritual resource that has been found to help family caregivers live through difficult transitions and challenges.

**Methods:**

Twenty-three participants from rural Western Canada completed daily journal entries documenting their hopes and challenges. Cortazzi’s (2001) method of narrative analysis was used to analyze the data, which was then transcribed into a narrative entitled ‘hope against hope.’

**Results:**

The journal entries highlighted: the caregivers’ hopes and what fostered their hope; the various challenges of caregiving; self-care strategies, and; their emotional journey. Hope was integrated throughout their entire experience, and ‘hope against hope’ describes how hope persists even when there is no hope for a cure.

**Conclusions:**

This research contributes to the assessment of caregiver interventions that impact hope and quality of life, while illustrating the value of a narrative approach to both research and practice. Journaling may be particularly valuable for rural caregivers who are isolated, and may lack direct professional and peer support. There is an opportunity for health professionals and other providers to foster a relationship of trust with family caregivers, in which their story can be told openly and where practitioners pay closer attention to the psychosocial needs of caregivers.

## Background

In palliative and end of life (P/EOL) care, much of the responsibility of caring for those who are dying rests on family caregivers, the majority of whom are women [1,2]. Caregivers in P/EOL care are particularly vulnerable to declines in their own health status, as they often prioritize the needs of the dying while neglecting their own. In a metasynthesis study of the hope experience of family caregivers with chronic illnesses, hope was defined as a psychological resource that helped them deal with their caregiving experience [3]. The hope of family caregivers was situational and influenced by relational and contextual factors. In a recent review of the literature on home-based family caregivers, Funk et al. suggested there is a need for research related to the relational and contextual influences on the family caregiver at the end of life [4]. The relational and contextual influences on the family caregivers are important to understand the caregiving experience and hope experience. Elucidating the stories of family caregivers is one way to capture these relational and contextual influences. When someone is nearing the end of their life, their caregiver is a witness to the person who is suffering and in pain [5]; the caregiver also experiences their own pain and suffering and has an interconnected, but distinct, story to tell. The purpose of this research is to share, in the form of a story, the experiences of rural female caregivers caring for family members with advanced cancer, focusing on what fosters their hope. The journals were collected as part of a larger research study on the hope of rural women caregivers of persons with advanced cancer.

This particular research paper focuses on the qualitative component of a mixed method study evaluating a hope intervention entitled Living With Hope Program (LWHP) [6]. The mixed method design employed was a concurrent explanatory design, with the emphasis on the quantitative data. Qualitative data informed the quantitative findings. Short, direct entries, written in participant journals, were collected as the qualitative component. The Living With Hope Program (LWHP) was designed to foster hope in caregivers of family members with advanced cancer; it was developed based on qualitative data [7] and from recommendations for family caregiver interventions [8]. The program was pilot tested for its feasibility, and was deemed to be not only feasible, but also flexible, easy to use, and showed promise in increasing the hope and quality of life of family caregivers [9].

The intervention of the LWHP consists of an international award-winning video, *Living with Hope* and a hope activity, entitled *Stories of the Present*, where participants complete a daily journal entry for two weeks, writing down their challenges and what gives them hope each day. Journaling allows individuals to cognitively organize stressful events, and provides a venue for reflection when daily life can be overwhelming and chaotic [10]. In the larger research study, the LWHP was given to rural female family caregivers of persons living with advanced cancer. The purpose of the in depth narrative analysis of the qualitative data collected from the journaling exercise provides an understanding of the experience of the participants while contributing to the context in which the LWHP is evaluated. In addition, it also identifies other factors that influence hope, all of which had an impact on the effectiveness of the LWHP.

Rural communities have an increasingly aging population and may be particularly vulnerable in P/EOL care [11]. Research on rural palliative care highlights how rural health services are fragmented, underfunded and lack specialists, and how caregivers are over-extended. Rural communities are also known for their resourcefulness and social cohesion, thus, there are strengths and challenges to rural P/EOL care provision [12,13]. This context emphasizes the vulnerability that these caregivers are experiencing amidst a critical time in their caregiving trajectory.

### Experience of caregiving

The negative physical, mental, emotional, social and economic consequences of providing care can be summarized into the term ‘caregiver burden.’ While most family caregivers want to be able care for their family member, they continue to experience caregiver burden and carry responsibilities beyond what they can handle physically and emotionally; this in turn negatively impacts their health and overall quality of life [14-16]. Caregiver burden can be exacerbated by the multiple roles and responsibilities that family caregivers have, including spouse, parent, and employee [17]. The common negative health outcomes that family caregivers experience include stress, anxiety, depression, sleep deprivation, fatigue, physical pain and other chronic health conditions [18-20]. Loneliness and fear can also be a part of the family caregiver’s experience, and the fear of the unknown is felt especially as the patient nears the end of their life [21,22]. Hope is a psychosocial and spiritual resource that has been found to help family caregivers in managing the challenges of caregiving.

### Caregiving and hope

Understanding the meaning and significance of hope and its relationship to quality of life has been a significant focus of research across disciplines and methodologies, specifically in literature related to health and illness. The meaning and processes of hope have been studied across a variety of health and illness experiences, including individuals living with a terminal illness [23,24], caregivers of persons living with chronic illness [3], caregivers of persons living with dementia [25,26], bereaved caregivers [27] and individuals living with HIV/AIDS [28].

As a psychosocial and spiritual resource, hope has been found to help family caregivers live through difficult transitions and challenges of the caregiving experience, and influences their quality of life [29,30]. Hope is related to how individuals behave, feel and think; it has been defined as an inner strength, as possibility for the future, and as a multidimensional, dynamic life force, among other descriptions. In a metasynthesis of the hope experience of family caregivers of persons with chronic illness [3], the authors found that hope involves the interrelated, coexisting themes of: transitional refocusing from a difficult present to a positive future; dynamic possibilities within uncertainty; different pathways of hope based on the degree of uncertainty, and; hope outcomes such as coping, decreasing grief and increasing well-being. This metasynthesis highlighted how hope is integrated with many aspects of the caregivers’ experiences, including the challenges and the uncertainty of caregiving. In the metasynthesis only two studies focused on the hope experience of family caregivers of persons with advanced cancer [7,31].

Borneman et al.’s study emphasized the importance of hope in the caregiving experience; common themes included the strong connection between hope and faith, and inter-relatedness with others [31]. Other themes included being realistically hopeful, taking things one day at a time, and hoping to decrease the patient’s suffering. Borneman et al. advocated for nurses to play a role in facilitating and maintaining hope in family caregivers. Holtsander et al.’s study of the experience of hope of family caregivers of palliative patients introduced a model for the hope pathway ‘Hanging onto Hope,’ which began with the palliative diagnosis [7]. There were experiences that could erode the hope of the caregiver, as well as experiences that fostered hope, such as relationships and spirituality. ‘Hanging onto Hope’ for the family caregivers involved writing their own story, staying positive, living in the moment, and doing what you have to do by accepting and not giving up. The participants described their hope as essential as it gave them the courage to continue to give care.

Not included in the metasynthesis study [3] as it was published later, in 2012, is an additional study of hope which used narrative analysis of journal entries of 10 caregivers to create a poem about the hope experience of family caregivers of someone with advanced cancer [32]. The intense chaos, filled with turbulence and uncertainty, as well as the daily search for hope, were apparent in the caregivers’ narratives. This study however, was the only published study that utilized a narrative approach to describe the experience of family caregivers of persons with advanced cancer; future research was recommended with larger sample size.

With only three published studies of hope among caregivers of persons with advanced cancer, there is a paucity of research in this area. Advanced cancer is distinguished from other end of life processes by the severity of its physical and psychosocial symptoms [33,34], which has an impact on the health and well being of caregivers [35]. The purpose this study was to describe the experience of family caregivers of persons with advanced cancer. The specific aims were to describe the experience of rural female caregivers caring for family member with advanced cancer, and to describe what fosters their hope.

## Methods

Increasingly, the importance of the narrative is being recognized in health care and more specifically in palliative care [36-39]. For example, by listening to patient and caregiver stories, care professionals can better understand and meet psychosocial and spiritual needs of those they are serving; by telling stories and having someone listen and respond the narrator can create new ways of seeing the world and their place in it [40]. Narrative is a term used to refer to structures of knowledge and storied ways of knowing [41]. The act of telling/writing may be therapeutic for the person doing the telling/writing; however, the purpose of this study was not to uncover the potential therapeutic nature of narratives. Most approaches to narrative in health care research focus on the patient experience; in P/EOL care, the patient and family is the unit of care. The stories of family caregivers must be engaged, as they are a constant witness to the experience of the dying person [6], and to their own experience of, suffering.

### Sample

Participants were recruited through palliative home care providers who identified potential participants based on the inclusion criterion and asked for their consent to provide their contact information to trained experience research nurses (RAs). Inclusion criterion were: 1) 18 years of older, 2) self-identify as rural, 3) female, and 4) living with and caring for a person with advanced cancer. If the potential participant agreed to be contacted, the RA contacted them and gave them information about the study. Rural women caregivers were the focus of this study as they are at most risk for the negative consequences of caregiving [6]. The number of participants was determined by those participants that wrote “Stories of the Present” in the evaluation study of the LWHP.

Twenty-three women living in rural Saskatchewan or Alberta participated in this study. The mean age of the participants was 59 years and of the care receiver 63.5 years. The relationship to the care receiver was primarily the spouse (n = 19), followed by daughter (n = 3) and mother (n = 1). The average amount of time spent caregiving prior to the study was thirty-four months.

### Data collection

Data collection began in January 2009 and was completed in March of 2012. Ethics approval was obtained from The Alberta Cancer Research Ethics Committee (#25209), the University of Saskatchewan Behavioural Research Ethics Board (#08-186), and the Regina Qu'appelle Health Region Research Ethics Board (REB-09-24).

After obtaining written consent, participants were given a journal entitled *Stories of the Present*, and asked to take five minutes each day to write down their challenges and what gave them hope. This protocol is detailed in the pilot study [6]. Three hundred and forty two journal entries were collected from 23 participants, and most of these entries were written daily for a two week period. A research assistant compiled the data and transcribed each entry, taking out any name and place identifiers.

### Data analysis

The transcribed data from the journals were checked for accuracy and was entered into the qualitative data management program NVivo 8. Narrative inquiry approach was used to analyze the journal data. It is an approach that values the human art of storytelling and allows the researcher to make sense of events and situations with multi-layered meanings, and to gain a rich and resonant insight and description of the lived experiences [36,37,41]. To provide a foundation to write the caregiver narrative, we drew upon Arthur Frank’s story types in his book *The Wounded Storyteller*, specifically the chaos narrative [42]. While many stories are written about triumph, or where meaning has been created out of suffering, the chaos narrative has no such triumph or sense of purpose; the chaos narrative is the place for those stories where there is no fixing and no way out. This applies to the caregiver experience in P/EOL care [32]. The chaos must be acknowledged before individuals can move forward and be able to tell their story.

Cortazzi’s method for narrative analysis was used to analyze the participant journals and provide a structure for the story that would emerge [41]. This method was chosen as it is consistent with the descriptive nature of the study purpose, as it seeks to systematically interpret other’s interpretation of events. Cortazzi divides stories into three narrative categories: the *event*, which is describing what happened or a series of events that happened; the *experience* which includes the feelings, reactions, images and meanings that the participant ascribes to the recounted events; and the *evaluation*, which is the broader interpretation of what happened and its significance. The first step of the analysis was thus to code the content of the journals into the three narrative categories. This process was also used in the pilot study of the LWHP, where the outcome of the analysis was in the form of a poem rather than a prose story [32].

The second step of the analysis was to conduct a thematic analysis of the journal entries within each of the three narrative categories (event, experience and evaluation). The main themes from the three categories were the expressed in the caregiver narrative The final step of the analysis was to incorporate the main themes of the data into one story, written in first person from a caregiver’s perspective, that expresses the caregiver’s hopes and challenges highlighting events in their life, their response to and evaluation of those events and the role that hope plays. To write the narrative, we used the principles of poetic transcription, which calls for staying true to the exact words of the participants as much as possible [32,43]. Having previously coded and sorted the data, statements and ideas from the journals were condensed and woven together into one whole story. Table 1 provides examples of the transcription process for each of the narrative categories. For each of the narrative categories, direct quotes from the journal data are given. Then in the final story the direct quotations representing the themes were highlighted. The final story that is presented below reflects the main themes in each category and utilizes direct quotations representing these themes as much as possible. Three authors were involved directly in the data analysis (AW, JE and WD). All three read the transcripts separately. JE began the initial analysis and then meet several times to discuss the analysis at each step. Consensus was reached at each step before proceeding.

**Table 1:** Narrative transcription examples of event, experience, evaluation

**Direct quotations from journals**	**Excerpt of narrative**
Journal *event*:	Transcribed into narrative:
Bad news – so bad it was hard to take in. A big growth in his lymph nodes that they did not see 4 weeks ago.	*A few weeks ago we received bad news that was hard to take in. When we saw the oncologist, he left us with the clear message that we are on a different path now that the cancer is back.*
When we saw Dr. D this morning, the radiation oncologist, he didn’t pull any punches & left us with the clear message that we have met a crossroad and now are on a different path that the cancer is back.	
Journal *experience*:	Transcribed into narrative:
It was both sobering and hard to hear. It was also hard because K does not verbalize his emotional response. He just keeps saying “What is IS and I completely accept it” whereas I am feeling of anger, sadness and fear… and shocked with the soberness.	*My partner is not showing emotion and says he accepts it, but I am feeling anger, sadness, and fear. I am still shocked with the soberness.*
Journal *evaluation*:	Transcribed into narrative:
To hope against hope is hoping even through there is no good reason to hope (which is where we were at today) is the kind of hope we have. No more can be done for my honey. That’s the sad reality we are facing.	*The kind of approach I currently have is “to hope against hope”, meaning you keep hoping even though there is no hope for a cure.*
That is the hope I have right now, see what lies ahead. There may be a light at the back of the tunnel yet.	*There may be light at the back of the tunnel yet – every once in a while it sneaks in when I’m not looking.*
There is hope. Every once in a while it sneaks in when I’m not looking.	

## Results

Participants’ journals focused on the challenges and hopes that they experienced daily, and also described what fostered their hope. Participants did not always tell a linear story with a beginning, middle and end; some focused specifically on daily activities and tasks, or fully on the state of the care receiver. Others structured their journals exactly according to their daily hopes and challenges; others were a mix of events, plans for the near and distant future, and the emotional experience. From the events, experiences and evaluations in the participant journals, the following four themes emerged to characterize the journals: *hope, practical and emotional challenges, self-care strategies, and the emotional journey*. We first illustrate how hope was described and experienced by the participants, followed by the remaining three that are interconnected with and influence hope, and vice-versa, and address the specific aim of what influences their hope contributing to the description of the event and experience.

### Hope

Hope, evidently, was one of the main themes that came through in the participant journals, as a significant goal of the journaling process was to document what gave them hope each day. Hope was described in many different ways; caregivers mentioned concrete, specific hopes they had for the immediate future; they described what hope meant to them; they shared what gave them hope; and they shared what took away their hope, or when they felt hopeless. One of the most common phrases that started or ended a journal entry was, “*I hope tomorrow is better.”*

Participants had specific hopes related to wanting the care receiver to feel better the next day, to not be in pain, hoping the weather is nice, hoping that therapy goes well, hoping for sleep and rest, for example, “My hope is that I can sleep tonight,” and, from another participant, “My hope for tomorrow is that [patient] will be stronger and eat better.” At the same time they wrote about a tension that often existed with their hopes, as “hoping against hope.” As one participant wrote: “To hope against hope is hoping even through there is no good reason to hope (which is where we were at today) is the kind of hope we have. No more can be done for my honey.” “Hoping against hope” is the tension between hoping for a cure for their family member and the recognition that this is no longer a possibility. At the same time they had other types of hope. Hope for these participants was also a *choice* and a *mindset*. Many women wrote about how they tried their best, despite extremely discouraging circumstances, to look for hope each and every day in the simple things, and in focusing on the present. They chose to look for the positive aspects of each day and also resorted to good memories of the lives they had lived with the care receiver. Choosing to be hopeful was not easy, and part of this mindset also involved acceptance of their circumstance, and striving to find a new ‘normal’ amidst so much uncertainty. The new normal and acceptance is described here by two participants:

“Now we have adapted to the new normal. We are still optimistic… I guess we both know the odds are not in our favour but it is hard to do anything else but push into it and hope for the best.”

“I know that we have changed our expectations of what is possible for our family and that’s ok. I think that my family has all adjusted to what our new “normal” is and we are trying to embrace our opportunities right now.”

Participants described several influences on their hope: temporal circumstances, social support, spirituality and faith.

### Circumstances

The *specific, temporal circumstances* of each day could foster or take away from the participants’ hope. Caregivers’ levels of hope and their general mood were heavily dependent on the mood of the care recipient and the care receiver’s state of health. Good days were more hopeful days. The weather and environment played a role in the caregivers’ perceptions of their hope, as one participant shared, “Hope, well it was hard to find any today except that the flowers are blooming beautifully, and that is something.” Factors that detracted from the participants’ hope were dreary weather, stressful travel and medical appointments, family conflict, and the declining health and well-being of the care receiver. One participant described her stress with driving and how she felt alone: “I feel alone even when people are here! Nobody really understands! [Care receiver] is sick. I need to lift him into the truck… I am scared of getting him into the truck tomorrow to go to [location of appointment].”

### Social support

*Social support* was found to be a key theme that fosters hope, whether this was support from family members, friends or health professionals such as doctors and nurses. Many caregivers mentioned how celebrating an occasion with family members, seeing their grandchildren, having extra support from their sons and daughters or their siblings, brought hope to their day and to their lives, for example, “Our friends and our health team give me hope for tomorrow… my support if & when [care receiver] needs them or we need them.” Loneliness, one of the emotional challenges of providing end of life care, can be combated through this social support. It must be noted that not all participants had supportive, positive family relationships which at times was the cause for additional strain and fatigue beyond the immediate caregiving experience. An example of this is one woman’s experience with her grown children: “I guess my nerves are frayed – I don’t seem to get too much support from my family – and I don’t have anyone to talk to and cry on their shoulder – my support person is dying – where is my hope?” Negative relationship dynamics took away from their hope.

### Faith

Some participants also found hope through their *faith*, and the belief that their fate was in the hands of God, or something bigger than themselves. They relied on prayer to get them through the day, and attributed their ability to cope and get through each day to their faith: “I must admit there are times when I question why so blinking much is on my plate and I have the faith of Job. However, even that tiny bit will get me through each day when I’m at a low ebb.” This was not always the case, and several participants could not reconcile God’s role in the suffering their family was experiencing, such as this woman: “I am not accepting of this – it can’t be God’s will – as he/she is supposed to provide love not pain – but again maybe even God has chosen to vent out his/her frustrations and I was handy…”

### Practical and emotional challenges

Participants extensively documented their various challenges in their journals, which were both practical and emotional. It was a challenge for participants to deal with the health struggles of the care recipient, and to complete the physical tasks of caregiving. Caregivers experienced the lack of time and energy to do everything they needed and to care for themselves. Family dynamics presented challenges, such as feeling controlled by their partner or feeling undermined and unappreciated by their children. These examples link back to the connection between hope and daily specific, temporal circumstances. An overarching challenge for the participants was the uncertainty, which presented a backdrop to every experience. Family caregivers realized that they, too, are vulnerable and have limitations; that they are not in control and cannot fix things on their own. This uncertainty was always present and could coexist with hope.

### Self-care strategies

Participants employed multiple self-care strategies to cope through some of the challenges and emotions of the caregiving experience. These included physical outlets, such as eating well, doing yoga, and running. Many participants kept busy in order to cope, and socialized with friends and family, such as going to dinner or for coffee. Some sought professional support such as counselling, and set goals for themselves. This links back to the connection between social support and hope. Participants also made a conscious effort to focus on the present and what they were grateful for; some allowed time for reflection in the form of prayer or journaling, supporting the notion that hope could be a choice and a mindset.

### Emotional journey

Participants wrote considerably about the emotional aspects of the caregiving experience, and it was evident that numerous emotions were at play throughout their journey, emotions that overlapped and sometimes contradicted each other. The emotional experience of the participants included fear, worry, sadness, guilt, helplessness, anger, loneliness, empathy, love and gratitude. Participants were generally fearful of the future, and of the uncertainty of the state of their loved ones and their lives. They expressed worry about specific things, such as how the care receiver would respond to treatment, the stress of travelling to medical appointments, the concern and guilt they felt anytime they were away from the care receiver. They expressed sadness around missing the way life used to be and the way their loved one used to be, and in imagining life without that person. Fear could detract from hope, while the love they gave and received contributed to their hope. The participants’ emotional journey speaks to the co-existence of hope and hopelessness, and strength and weakness, in the caregiver experience, and how hope is a multi-layered phenomenon. Participants continued to hope and chose to hope despite knowing there was no cure for the care receiver’s illness.

### The story

Frank [42,44] writes that a story can only be told in the context of a relationship, a dialogical relationship between the teller and listener. The researcher or analyst is a part of the relationship that a story asks for, as a listener and a witness, and any methodology we use must follow the ethical commitment of living and telling stories for the other, as *“to tell any story of suffering is to claim some relation to the inter-human”* (42, p. 180). We now present the story that is the outcome of the narrative analysis of the journals reflecting the themes presented above. It is entitled ‘Hope against Hope’ to depict the type of hope that many of the participants were experiencing while providing care. The bolded statements correlate to Table 1 showing how the themes in each of the categories are represented in the narrative.

Hope against hope

*The initial cancer diagnosis was just over a year ago – wow, we have been through a life-changing journey. We have both journeyed through diagnosis, surgery, treatment, recovery, myself going with him to every appointment, going back and forth from the city to home. A few weeks ago we received bad news that was hard to take in. When we saw the oncologist, he left us with the clear message that we are on a different path now that the cancer is back. My partner is not showing emotion and says he accepts it, but I am feeling anger, sadness, and fear. I am still shocked with the soberness. I know that the Doctor and his team are trying,* ***but it is hard to know what to feel. I am scared to get my hopes up***.

*Often, I am too tired to think about how I feel. Starting this journal has turned my focus within and that journey with self has been a bit rough. The busyness of life has not given me enough time to process all of what we've been through this past year. I am writing this at midnight as it seems to be the only time for me. I have no life outside of cooking, cleaning, laundering, driving into town for medical appointments, together with the constant focus on how my partner is doing. I sometimes feel very lonely living in this little ‘world’ of ours. Although receiving company is good for us, sometimes I don’t want to see anyone.* ***I try to be the best person I can be, but sometimes it is hard to find the strength to do that****.I am looking forward to seeing an old friend for coffee tomorrow, who has kind of been through the same thing. It will be good to talk to her. Our son is coming down to take care of my partner so I do not have to worry. I still feel guilty though, for leaving him. But I know I need time for myself.*

*I am having more difficulty now than I have over the past few months. My mood depends on how my partner is doing. I am tired; I need a break.* ***I am losing my grip on my future; the unknown scares me and hangs over my head****. Everyone says to live one day at a time but it is hard when your mind is racing with all the things to do, in addition to being constantly reminded that there is no hope; it is overwhelming.* ***My support person is dying - I just want to be able to fix things and that’s not going to happen****. I want my old life back. I am trying to adjust to a new “normal” but it is hard to find the balance; I know I am very vulnerable right now. My feelings are raw.*

*The kind of approach I currently have is* ***“to hope against hope”, meaning you keep hoping even though there is no hope for a cure****. I try to focus on living life to the fullest more than on dying. I am committed to my partner and he won’t go through this alone as long as I can dare these sore tired feet to carry me that extra mile. I need to be strong for him. Somehow, even a tiny bit of faith will get me through each day when I’m feeling low. Sunny and warm weather helps our moods. I am grateful for my family and friends. When there is laughter, that gives me hope. When we see our beautiful grandchildren, they give me hope. I guess I need to look for hope every day because it is the one part of the disease that I can control, unlike how the cancer progresses – that is a challenge as we wish we could do something about that the most.* ***But I can choose to hope. There may be light at the back of the tunnel yet – every once in a while it sneaks in when I’m not looking.***

## Discussion

What we learned about fostering hope from the participant journals bears many similarities with the sub-processes of the ‘hanging onto hope’ pathway [26]: supporting relationships (social support), connecting with something bigger (faith), writing your own story (reflection), staying positive (mindset), living in the moment (focus on the present), doing what you have to do (acceptance). These concepts were also described in a metasynthesis study of the hope experience of family caregivers of persons with chronic illness [3].

The concept of ‘hope against hope’ has not been previously found in the research on hope and family caregivers. As such it can enhance the ‘hanging onto hope’ model, in that it illustrates how caregivers persevere and continue to find hope when possibilities for the future have not yet been recognized. “Hoping against hope” has been described in a conceptual analysis of hope as a typology of hope [45]. It reflected the continuous process of hoping to get through different barriers that repeatedly emerged by women with breast cancer. Our study findings however, suggest that “Hoping against hope” was descriptive of the hope experience of family caregivers of persons with advanced cancer. This concept was related to the tension of hope for a cure and hope for peace and comfort at the end of life. The tension was also described in a study of persons at the end of life [46]. Further research is required to determine if this concept is similar to that presented in the typology.

The concept of “hoping against hope” highlights how seemingly contradictory experiences and emotions can coexist: caregivers chose to hold onto their hopes no matter what the circumstances, but this also coincided with feelings of fear, loneliness and hopelessness. Although hope is a very different concept than coping [47] the dynamic nature of “hoping against hope” maybe similar to the tension and dynamic between coping and resilience in caregiving versus the effects of caregiver burden is documented in other research [22,48]. This however requires further research.

Each of the caregivers who participated in this study are on their own unique journeys, and the narrative of ‘hope against hope’ demonstrates a thread that brings their stories together and allows us as researchers and those who read this story to bear witness to their experiences. In bearing witness to the caregiver narrative(s), we must acknowledge and honour the chaos that is within it; that there are days and periods of time when it seems that there is no way forward, that there is no purpose, that there is no longer any hope. As Frank writes, *“Chaos is never transcended but must be accepted before new lives can be built and new stories told”* (42, p. 110)*.* When one is inside the chaos, they are not yet in a place to be able to articulate and share what they are going through; there is no language to match the experience. Reflective space is required to emerge from the chaos; the journaling provided that space of reflection, and caregivers used it to both answer the precise questions of the study (what fosters hope and what are their challenges), and to write down whatever was in their hearts and minds at the time. The process allowed for individuals to cognitively reframe their experience in order to maintain hope and find meaning out of the chaos [49].

The story of ‘hope against hope’ , and the themes that emerged from the participant journals highlights a need for psychosocial, peer, and bereavement support for family caregivers, which has also been recommended in previous research [50-52]. One participant described the need to connect with a “soul sister” who is on a similar journey. Other participants wrote about how they do not have the time or energy to reach out for their own support, but were nevertheless overwhelmed by the emotional burden of P/EOL care. Some wrote about the difficulty of change in their partnership. Others struggled with coming to grips with the idea of life without the care receiver. Journaling and a narrative approach in general is a step forward in providing support; a strength of the LWHP is that it allows those who can benefit from reflection of this kind to so without requiring professional facilitation. Journaling as a therapeutic strategy has been long recognized [53] and may be particularly relevant in rural communities, where caregivers and their families experience various forms of isolation given their geographical location; this includes less contact with health professionals/service providers and less peer support. To make the *Stories of Hope* resource accessible to more people, we plan to make it available on the LWHP website and on the website of the Canadian Hospice Palliative Care Association.

### Limitations

One of the foremost limitations in this work is the study design. The particular narrative approach utilized in the study was descriptive in nature because of the limitation of the overall study design and data collection method. For example, the data was collected from a mixed method study, where the emphasis was quantitative; thereby the data was collected within a positivist study paradigm. The study design limited the co-construction of knowledge between the participant and researcher and, as such, future research should include an interpretive design with in-depth qualitative interviews. Related to this, a further limitation is that the ‘hope against hope’ story presented in the results did not and could not encompass all of the different expressions of the participants’ journey and their hopes and challenges. “ Stories of the Present” limited the act of journaling by providing instructions to take only 5 minutes at the end of each day and what was to be included. The findings then are a description of the experience of caregivers and what fosters their hope, which is consistent with the study purpose, but limits a more in-depth analysis of the possible therapeutic nature of this activity. Future research may include the use of “Stories of the Present”, coupled with in-depth qualitative interviews to understand possible benefits from this type of narrative.

Every experience is unique, and caregivers experienced hope or a lack of hope in different ways. While we performed a rigorous analysis of journal data, the story that was written as an outcome of the analysis was not verified by the participants. Although this was never intended as a part of the process, it should be considered for future research to improve rigour. Participants did receive a report documenting the results of the larger study. Another limitation is that the structure of the LWHP likely influenced the content of the journals, as it included watching the *The Living with Hope* video and being asked directly to write about hopes and challenges. *The Living with Hope* video features family caregivers and palliative patients’ discussion of hope and how they maintain hope. It is 15 minutes in length and was shown after baseline data was collected in the study. It is not known if this film had an influence on the content of the journals. Thus, journals may have been more ‘hopeful’ than if the caregivers had journaled without such prompts.

This study is also limited due to the specificity of the targeted group of participants, who were female caregivers living in rural Western Canada, caring for a family member with advanced cancer. The literature on hope is inconclusive, with respect to whether women interpret or view hope differently than men. The caregiving experience may be different for men than women [54], so the findings of our study may not be applicable to men caregivers. However this research brings forward a novel illustration of how hope is integrated throughout the caregiver’s experience.

## Conclusions

This study has explored the hopes and challenges of rural female family caregivers of persons with advanced cancer. The journal entries participants completed as part of the LWHP provided insight into their daily lived experience, and highlighted their emotional journey, the various challenges of caregiving, the way they employed self-care strategies, the various hopes they had and what fostered their hope. The concept of “hoping against hope” highlights the existence of tension and the possible co-existing contradictions with hope. At the same time that the participants are hoping for a cure, they are also hoping for their family member to have peace and comfort at the end of life. This research contributes to the much-needed assessment of P/EOL caregiver interventions, specifically those that impact hope and quality of life, and illustrates the value of a narrative approach to both research and practice [37]. ‘Hope against hope’ calls for researchers, health professionals and other supports in P/EOL care to encourage self-care strategies, self-reflection and social support to enhance caregivers’ hope and capacity to cope while caring for someone with a terminal illness. There is an opportunity for health professionals and other P/EOL care supports in various settings to foster a relationship of trust with family caregivers in which their story can be told openly and in which they are holistically supported.

## Abbreviations

P/EOL: Palliative/end-of–life; LWHP: Living with hope program.

## Competing interests

The authors declare that they have no competing interests.

## Authors’ contributions

WD, AW, RT, DC, and LH conceptualized the study and obtained funding. WD, as nominated PI, was responsible for the overall study coordination including recruitment, data collection and transcription of the data. AW (Co-PI) was responsible for the analysis of the data. JE, AW and WD analyzed the journal entries. JE wrote the initial draft of the manuscript in ongoing and close consultation with AW. JE met with AW and WD several times to discuss the analysis. All authors contributed to the manuscript by submitting comments and suggestions. All authors read and approved the final manuscript.

## Pre-publication history

The pre-publication history for this paper can be accessed here:

http://www.biomedcentral.com/1472-684X/12/44/prepub

## References

[B1] Williams A Crooks VA Introduction: space, place, and the geographies of women’s caregiving work Gender, Place and Culture 2008 12 3 243 247 10.1080/09663690801996254

[B2] Carstairs S Raising the bar: A roadmap for the future of palliative care in Canada 2010 Senate of Canada: Ottawa

[B3] Duggleby W Holtslander L Kylma J Duncan V Hammond C Williams A Metasynthesis of the hope experience of family caregivers of persons with chronic illness Qual Health Res 2010 12 2 148 158 10.1177/1049732309358329 20065303

[B4] Funk L Stajduhar K Toye C Grande GE Todd CJ Part 2: Home-based family caregiving at the end of life: a comprehensive review of published qualitative research (1990–2008) Palliat Med 2010 12 6 594 607 10.1177/0269216310371411 20576673

[B5] Ferrell B Ethical perspectives on pain and suffering Pain Manag Nurs 2005 12 3 83 90 10.1016/j.pmn.2005.06.001 16129379

[B6] Duggleby W Williams AM Living with hope: developing a psychosocial supportive program for rural women caregivers of persons with advanced cancer BMC Palliative Care 2010 12 3 Accessed from http://www.biomedcentral.com/1472-684X/9/3/10.1186/1472-684X-9-3 20346156 PMC2859076

[B7] Holtslander L Duggleby W Williams AM Wright K The experience of hope for informal caregivers of palliative patients J Palliat Care 2005 12 4 285 291 16483098

[B8] Harding R Higginson IJ What is the best way to help caregivers in cancer and palliative care? A systematic literature review of interventions and their effectiveness Palliat Med 2003 12 63 74 10.1191/0269216303pm667oa 12597468

[B9] Duggleby W Wright K Williams AM Degner L Cammer A Holtslander L Developing a Living with Hope Program for caregivers of family members with advanced cancer J Palliat Care 2007 12 1 24 31 17444459

[B10] Pennebaker J Seagal J Forming a story: the health benefits of narrative J Clin Psychol 1999 12 10 1243 1254 10.1002/(SICI)1097-4679(199910)55:10<1243::AID-JCLP6>3.0.CO;2-N 11045774

[B11] Castleden H Crooks V Schuurman N Hanlon L “It’s not necessarily the distance on the map…”: Using place as an analytic tool to elucidate geographic issues central to rural palliative care Health Place 2010 12 284 290 10.1016/j.healthplace.2009.10.011 20005147

[B12] Butler SS Turner W Kaye LW Ruffin L Downey R Depression and caregiver burden among rural elder caregivers J Gerontol Soc Work 2005 12 1 47 63 10.1300/J083v46n01_04 16338884

[B13] Robinson CA Pesut B Bottorff JL Issues in rural palliative care: views form the countryside Journal of Rural Health 2010 12 1 78 84 10.1111/j.1748-0361.2009.00268.x 20105272

[B14] Cohen SR Leis AM Kuhl D Charbonneau C Ritvo P Ashbury FD QOLLTI-F: measuring family carer quality of life Palliat Med 2006 12 755 767 10.1177/0269216306072764 17148530

[B15] Mangan PA Taylor KL Yabroff R Fleming DA Ingham JM Caregiving near the end of life: Unmet needs and potential solutions Palliative and Supportive Care 2003 12 3 247 259 16594425 10.1017/s1478951503030414

[B16] Schulz R Mendelsohn AB Haley WE Mahoney D Allen RS Zhang S Thompson L Belle SH End-of-life care and the effects of bereavement on family caregivers of persons with dementia N Engl J Med 2003 12 20 1936 1942 10.1056/NEJMsa035373 14614169

[B17] Clemmer SJ Ward-Griffin C Forbes D Family members providing home-based palliative care to older adults: the enactment of multiple roles Can J Aging 2008 12 3 267 283 10.3138/cja.27.3.285 19158043

[B18] Carretero S Garcés J Ródenas F Sanjose V The informal caregiver’s burden of dependent people: Theory and empirical review Arch Gerontol Geriatr 2009 12 1 74 79 10.1016/j.archger.2008.05.004 18597866

[B19] Ostwald SK Who is caring for the caregiver? Promoting spousal caregiver’s health Family and Community Health 2009 12 S1 S5 S14 19065094 10.1097/01.FCH.0000342835.13230.a0

[B20] Schulz R Sherwood PR Physical and mental health effects of family caregivers Am J Nurs 2008 12 9 Supplement 23 27 18797217 10.1097/01.NAJ.0000336406.45248.4c PMC2791523

[B21] Aoun SM Kristjanson LJ Currow DC Hudson PL Caregiving for the terminally ill: at what cost Palliat Med 2005 12 551 555 10.1191/0269216305pm1053oa 16295288

[B22] Proot IM Abu-Saad HH Crebolder HFJM Goldsteen M Luker KA Widdershoven GAM Vulnerability of family caregivers in terminal palliative care at home; balancing between burden and capacity Scand J Caring Sci 2003 12 113 121 10.1046/j.1471-6712.2003.00220.x 12753511

[B23] Duggleby W Wright K Transforming hope: how elderly palliative patients’ live with hope Can J Nurs Res 2005 12 2 70 84 16092779

[B24] Wennman-Larsen A Tishleman C Advanced home care for cancer patients at the end of life: a qualitative study of hopes and expectations of family caregivers Scand J Caring Sci 2002 12 240 247 10.1046/j.1471-6712.2002.00091.x 12191035

[B25] Duggleby W Williams AM Wright K Bollinger J Renewing everyday hope: family caregivers of persons with dementia Issues Ment Health Nurs 2009 12 8 514 521 10.1080/01612840802641727 19591026

[B26] Irvin BL Acton GJ Stress, hope, and well-being of women caring for family members with Alzheimer’s disease Holist Nurs Pract 1997 12 2 69 79 10.1097/00004650-199701000-00010 9035623

[B27] Holtslander L Duggleby W The hope experience of older bereaved women who cared for a spouse with terminal cancer Qual Health Res 2009 12 388 400 10.1177/1049732308329682 19224881

[B28] Kylma J Dynamics of hope in adults living with HIV/AIDS: a substantive theory J Adv Nurs 2005 12 6 620 630 10.1111/j.1365-2648.2005.03633.x 16313375

[B29] Herth K Hope in the family caregiver of terminally ill people J Adv Nurs 1993 12 538 548 10.1046/j.1365-2648.1993.18040538.x 8496501

[B30] Duggleby W Swindle J Peacock S Ghosh S A mixed methods study of hope, transitions, and quality of life in family caregivers of persons with Alzheimer’s disease BMC Geriatr 2011 12 88 10.1186/1471-2318-11-88 22192235 PMC3268103

[B31] Borneman T Stahl C Ferrell B Smith D The concept of hope in family caregivers of cancer patients at home J Hosp Palliat Nurs 2002 12 1 21 33 10.1097/00129191-200201000-00012

[B32] Duggleby W Williams AM Holtslander L Cunningham S Wright C The chaos of caregiving and hope Qual Soc Work 2012 12 5 459 469 10.1177/1473325011404622

[B33] Duat R Cleeland C The prevalence and severity of pain in cancer Cancer Pract 1982 12 1913 1918 10.1002/1097-0142(19821101)50:9<1913::aid-cncr2820500944>3.0.co;2-r 7116316

[B34] Foley K Arbit E Scope of the cancer pain problem Management of Cancer Related Pain 1993 Mount Kisco, NY: Futura Publishing Company, INC

[B35] Valdimardsottir U Helgason AR Furst C-J Adolfsson J Steineck G The unrecognized cost of cancer patients' unrelieved symptoms: A nationwide follow-up of their surviving partners Br J Cancer 2002 12 1540 1545 10.1038/sj.bjc.6600271 12085201 PMC2746591

[B36] Bingley AF Thomas C Brown J Reeve J Payne S Developing narrative research in supportive and palliative care: the focus on illness narratives Palliat Med 2008 12 653 658 10.1177/0269216308089842 18612032

[B37] Charon R Narrative medicine: a model for empathy, reflection, profession and trust JAMA 2001 12 15 1897 1902 10.1001/jama.286.15.1897 11597295

[B38] Sandelowski M We are the stories we tell: narrative knowing in nursing practice J Holist Nurs 1994 12 1 23 33 10.1177/089801019401200105 7806848

[B39] Thomas C Reeve J Bingley A Brown J Payne S Lynch T Narrative research methods in palliative care contexts: two case studies J Pain Symptom Manage 2009 12 5 788 796 18954961 10.1016/j.jpainsymman.2008.05.006

[B40] Southall DJ Creating new worlds: the importance of narrative in palliative care J Palliat Care 2011 12 4 310 314 22372286

[B41] Cortazzi M Atkinson P, Coffey A, Delamont S, Lofland J, Lofland L Narrative analysis in ethnography Handbook of ethnography 2001 Oaks (CA): SAGE 384 394

[B42] Frank A The Wounded Storyteller: Body, Illness, and Ethics 1995 Chicago: University of Chicago Press

[B43] Glesne C That rare feeling: representing research through poetic transcription Qual Inq 1997 12 2 202 221 10.1177/107780049700300204

[B44] Frank A The standpoint of storyteller Qual Health Res 2000 12 3 354 365 10.1177/104973200129118499 10947481

[B45] Morse J Doberneck B Delineating the concept of hope J Nurs Scholarsh 1995 12 4 277 285 10.1111/j.1547-5069.1995.tb00888.x 8530115

[B46] Benzein E Norberg A Savemen B The meaning of the lived experience of hope in patients with cancer in palliative home care Palliat Med 2001 12 117 226 10.1191/026921601675617254 11301662

[B47] Folkman S Carr BI, Steel J Stress, Coping and Hope Psychological Aspects of Cancer 2013 New York: NY: Springer 119 127

[B48] Stajduhar K Martin WL Barwucg D Fyles G Factors influencing family caregivers’ ability to cope when providing end of life cancer care at home Cancer Nurs 2008 12 1 77 85 10.1097/01.NCC.0000305686.36637.b5 18176135

[B49] Neimeyer RA Reauthoring life narratives: grief therapy as meaning reconstruction Israel Journal of Psychiatry 2001 12 171 183 11725416

[B50] Grbich C Parker D Maddocks I The emotions and coping strategies of family members with a terminal cancer J Palliat Care 2001 12 1 30 36 11324182

[B51] Henriksson A Benzein E Ternestedt B Andershed B Meeting needs of family members of persons with life-threatening illness: a support group program during ongoing palliative care Palliative and Supportive Care 2001 12 263 271 10.1017/S1478951511000216 21838947

[B52] Rabow M Hauser JM Adams J Supporting family caregivers at the end of life: "they don't know what they don't know" JAMA 2004 12 4 483 491 10.1001/jama.291.4.483 14747506

[B53] Senn LC The Many Faces Of Journaling: Topics &: Techniques For Personal Journal Writing 2008 Saint Louis, MO: Pen Central Press

[B54] Giesbrecht M Crooks VA Williams A Hankivsky O Critically examining diversity in end-of-life family caregiving: implications for equitable caregiver support and Canada’s Compassionate Care Benefit Int J Equity Health 2012 12 65 82 10.1186/1475-9276-11-65 23116474 PMC3502300

